# Prognostic implications of unrecognized myocardial infarction and periprocedural myocardial injury on cardiac magnetic resonance imaging in patients with chronic coronary syndrome

**DOI:** 10.1038/s41598-023-40883-2

**Published:** 2023-08-21

**Authors:** Yoshihisa Kanaji, Masahiro Hoshino, Masahiro Hada, Ilke Ozcan, Tomoyo Sugiyama, Kazuki Matsuda, Kodai Sayama, Kai Nogami, Tatsuhiro Nagamine, Yun Teng, Toru Misawa, Makoto Araki, Eisuke Usui, Tadashi Murai, Taishi Yonetsu, Tetsuo Sasano, Tsunekazu Kakuta

**Affiliations:** 1 Division of Cardiovascular Medicine, Tsuchiura Kyodo General Hospital, 4-1-1, Otsuno, Tsuchiura, Ibaraki 300-0028 Japan; 2 Department of Cardiovascular Medicine, Mayo Clinic, Rochester, MN USA; 3 Department of Cardiovascular Medicine, Tokyo Medical and Dental University, Tokyo, Japan

**Keywords:** Interventional cardiology, Outcomes research, Cardiology, Medical research

## Abstract

This study sought to evaluate the prognostic implications of the presence of preprocedural unrecognized myocardial infarction (UMI) and periprocedural myocardial injury (PMI) evaluated by delayed gadolinium enhancement cardiac magnetic resonance (DE-CMR) in patients with chronic coronary syndrome (CCS) undergoing elective percutaneous coronary intervention (PCI). We enrolled 250 CCS patients scheduled for elective PCI. UMI was defined as the presence of late gadolinium enhancement (LGE) detected by pre-PCI CMR in the region without medical history of revascularization and/or MI. Periprocedural new occurrence or increased volume of LGE in the target territory detected by post-PCI CMR (PPL) were used to assess PMI. In the final analysis of 235 patients, UMI and PPL were detected in 43 patients (18.3%) and 45 patients (19.1%), respectively. During follow-up for a median of 2.2 years, major adverse cardiac events (MACE) occurred in 31 (13.2%) patients. On multivariable analysis, UMI and PPL remained as significant predictors of MACE after adjusting confounding factors (HR 4.62, 95% CI 2.24–9.54, P < 0.001, HR 2.33, 95% CI 1.11–4.91, P = 0.026). In patients with CCS who underwent elective PCI, UMI and PPL were independent predictors of worse outcomes. UMI and PPL on DE-CMR might provide additional potential insight for the risk stratification of patients undergoing elective PCI.

## Introduction

Percutaneous coronary artery interventions (PCI) are routinely indicated as a choice of treatment in patients with chronic coronary syndrome (CCS). However, invasive strategies were shown to be ineffective in reducing ischemic events and improving survival in patients with CCS, raising debate on the value of PCI in this population. A variety of patient and lesion characteristics and periprocedural factors have been suggested to be associated with worse outcomes in CCS patients undergoing PCI, and recognition of these factors might provide important prognostic value and pre-procedural risk stratification.

A non-negligible proportion of all acute myocardial infarction (MI) is asymptomatic or atypically presented without clinical recognition^1,2^. Previous studies have indicated that up to one-third of patients with CCS have previously complicated unrecognized myocardial infarction (UMI)^1^ and that UMI can carry equally or worse prognosis than clinically recognized myocardial infarction^3,4^. Periprocedural myocardial injury (PMI) that might occur during PCI has also been reported to be associated with worse outcomes in patients with CCS undergoing revascularization. However, the prognostic importance of periprocedural myocardial injury (PMI) related to elective revascularization still remains to be established due to controversies regarding the optimal definitions for the diagnosis of prognostically meaningful PMI^5^.

Delayed enhancement CMR imaging (DE-CMR) can noninvasively detect even minute myocardial scar presumably caused by coronary plaque rupture, coronary plaque erosion, coronary spasm, or other mechanisms including microvascular dysfunction that either spontaneously reperfused or that was nonocclusive^6,7^. Recent studies reported that the detection of infarct by DE-CMR is more accurate than clinical history, electrocardiography (ECG), or other imaging modalities^8^. Furthermore, periprocedural new late gadolinium enhancement (LGE) detected by post-PCI CMR might show better prognostic value than biomarker-evaluated PMI^9^. However, the clinical importance of UMI and PMI evaluated by pre- and post-PCI DE-CMR in the same patient cohort and the relative impact of these pre- and post-procedural infarcts on outcomes in patients with CCS remain elusive. Thus, the present study sought to investigate the relevance and prognostic value of the presence of UMI and PMI evaluated by serial DE-CMR examinations in patients with CCS undergoing elective PCI.

## Methods

### Study population

This is a substudy of the prospective study in which protocol was previously reported^10^. Briefly, this study was conducted at Tsuchiura Kyodo General Hospital between May 2017 and April 2020. Patients with CCS who were scheduled to undergo elective PCI were prospectively enrolled, and serial evaluations by DE-CMR before and after PCI were performed. These patients were selected from our regular clinical population based on the following inclusion criteria: age > 20 years, detection of an identifiable, single culprit lesion located in the proximal portion of a native coronary artery, and symptomatic ischemia or objective ischemia according to non-invasive stress testing including exercise tests, stress scintigraphy, perfusion cardiac magnetic resonance (CMR) imaging, and fractional flow reserve (FFR) measurements. CCS was defined by consistent frequency, duration, or intensity of anginal symptoms within 6 weeks before PCI. The target lesion was identified based on the combination of coronary angiography, electrocardiographic findings, noninvasive stress testing, and/or FFR values. The exclusion criteria included angiographically significant left main coronary artery disease, significant valvular disease, previous coronary artery bypass surgery, significant arrhythmia, renal insufficiency with a baseline serum creatinine level of > 1.5 mg dL^−1^, and contraindication to CMR (e.g., pacemaker, internal defibrillator or other incompatible intracorporal foreign bodies, pregnancy, severe renal dysfunction, and claustrophobia). Patients with impaired systolic function (< 50%) were also excluded. Prompt optional medical therapy was initiated in all patients after enrollment before PCI. This study was conducted in accordance with the guidelines and approval (TKGH-IRB 2017#628) to the Institutional Ethics Committee of Tsuchiura Kyodo General Hospital. The present study also complied with the Declaration of Helsinki for investigation in human beings, and all patients provided written informed consent before enrollment.

### Invasive coronary angiography and interventions

Each patient initially underwent standard selective diagnostic coronary angiography via radial artery using 5Fr system to assess the coronary anatomy. Quantitative coronary angiography analyses were performed using a CMS-MEDIS system (Medis Medical Imaging Systems, Leiden, The Netherlands). PCI procedure was performed using a 6Fr system via radial artery as a staged procedure after pre-PCI DE-CMR. Target lesions were physiologically assessed by fractional flow reserve (FFR) unless these had more than 90% stenosis. All patients underwent coronary drug-eluting stent implantation with predilatation. The type of stent (newer than the second-generation drug-eluting stent) was selected at the operator’s discretion, and the strategy was determined by the interventionist. Patients were maintained on dual antiplatelet therapy for at least 3 months after PCI.

### CMR image acquisition

After diagnostic CAG, eligible patients underwent DE-CMR image acquisition using a 1.5-Tesla scanner (Philips Achieva, Philips Medical Systems, Best, the Netherlands) with 32-channel cardiac coils both pre-PCI and post-PCI within 1 month to evaluate serial changes. Details are described in the Supplemental Material.

### CMR image analysis

Analysis of CMR images was performed using proprietary software (Philips Achieva, Philips Medical Systems, Best, The Netherlands) in a blinded fashion by two experienced investigators (Y.K. and K.N.), with the addition of a third one (T.K) when consensus was not obtained initially.

Ventricular volumes, ejection fraction, and LV mass were derived by contouring endo- and epicardial borders on the short-axis cine images. LV mass and volumes were calculated from Simpson’s rule using CMR data^11^. The presence of MI was assessed on DE-CMR images by identifying regions of contrast enhancement with an ischemic distribution pattern as subendocardial or transmural hyperenhancement. The amount of LGE was assessed using a threshold of 5 standard deviations above the signal intensity of normal myocardium as proposed by current recommendations^12^. UMI was defined as the presence of LGE detected by pre-PCI CMR in the lesion without a medical history of revascularization and/or MI, and PMI was assessed by a new occurrence or increased volume of LGE in the target territory after revascularization (PPL) in this study. To assess the occurrence of periprocedural new or additional LGE adjacent to or overlapping with UMI of target territory infarct after revascularization, pre-PCI and post-PCI scans were read side by side in all cases to assess the difference between UMI and PPL.

### Cardiac biomarker elevation after PCI

Blood samples for serial measurements of high-sensitivity cardiac troponin I level (hs-cTnI) and creatine kinase-MB (CK-MB) were collected before and 6, 12, 24, 48 h after PCI completion. Hs-cTnI was measured using the ARCHITECT i2000SR STAT hs-cTnI assay (Abbott Laboratories, North Chicago, IL, USA); the lower limit of detection was 1.5 ng L^−1^ and the 99th percentile upper reference limit (URL) is 26 ng L^−1^ according to the test obtained in healthy population with cardiovascular risk < 10%.

### Assessment of outcomes

Patients were followed up for the occurrence of MACE, defined as cardiac death, nonfatal MI, hospitalization for congestive heart failure, stroke, and unplanned late revascularization requiring hospitalization after the index PCI. Cardiac death was defined as any death preceded by acute valvular regurgitation, decompensated heart failure, ventricular arrhythmia, or new acute MI or death preceded by mechanical complications of MI. Clinical endpoints were determined by the blinded assessment of hospital records or via telephone interviews. Time to event was calculated as the period between pre-PCI CMR study and the first occurrence of MACE. Patients without MACE were censored at the time of the last follow-up.

### Statistical analysis

Statistical analysis was performed using SPSS version 25.0 (SPSS, Inc., Chicago, IL, USA) and R version 4.1.2 (The R Foundation for Statistical Computing, Vienna, Austria) software. Categorical data are expressed as absolute frequencies and percentages and were compared by the χ^2^ or Fisher’s exact tests. Continuous variables are expressed as mean ± standard deviation for normally distributed variables or as median (25th–75th percentile) for non-normally distributed variables and were compared using Student’s *t-*tests and the Mann–Whitney U-test, respectively. Qualitative variables were compared using Cohen’s kappa (κ) to quantify the level of agreement between infarct size assessment by cardiac biomarkers and DE-CMR. Survival curves were estimated using Kaplan–Meier estimates and were compared using log-rank tests. A Cox proportional hazards regression model was used to identify independent predictors of MACE. In the multivariate analysis, model 1 was adjusted for age and sex, and model 2 was adjusted for the covariates with P < 0.05. A collinearity index was used for checking linear combinations among covariates, and Akaike information criterion for avoiding overfitting. A two-sided P < 0.05 was considered statistically significant.

## Results

### Baseline patient characteristics, angiographical and CMR findings

**Figure 1:**
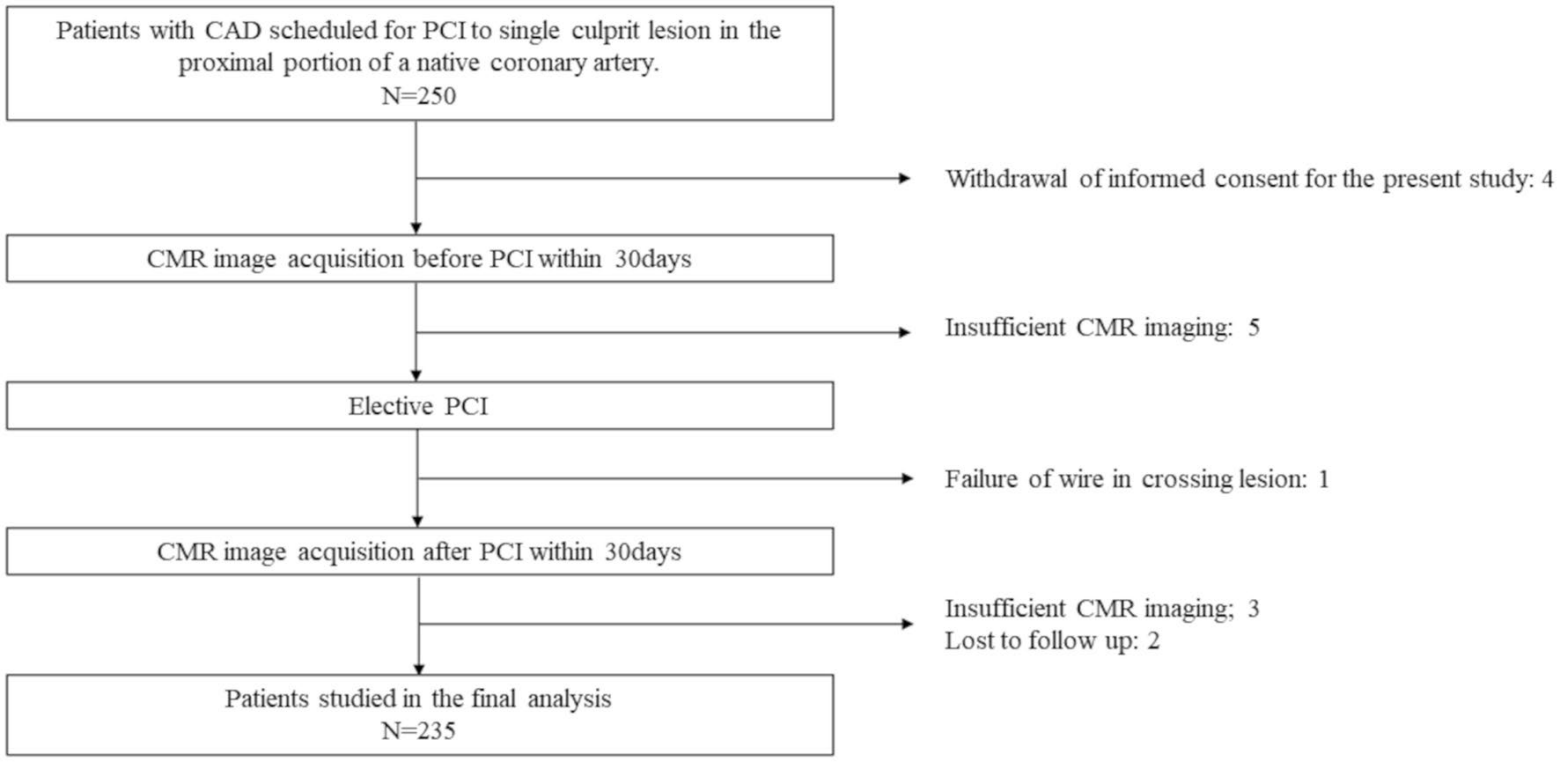
Study flow chart. Figure shows the enrollment process with a total of 235 patients in the final analysis.

Of 250 initially enrolled patients, the final analysis was conducted on 235 patients (Fig. 1). Patients underwent pre-PCI CMR within a median of 4 (2–7) days before PCI and post-PCI CMR within a median of 12 (7–18) days after PCI completion. No significant CMR-related complications were observed and stent implantation was performed successfully in all patients for the final analysis. Baseline patient characteristics, angiographic and CMR findings of 235 patients are summarized in Table 1, and the detailed data set according to the presence or absence of UMI and PPL are shown in Supplemental Table 1. The mean age of the study population was 67 years, 15.3% were women, and the median left ventricular ejection fraction (LVEF) was 62 (54–68) %. A total of 40 (17.0%) patients had a prior history of MI. Compared to patients without UMI, those with UMI were less likely to have a history of MI (P = 0.017, respectively), whereas there was no significant difference in the prevalence of prior history of MI between the patients with and without PPL (P = 0.14, respectively).

**Table 1:** The baseline clinical characteristics.

	Total, N = 235
Demographics	
Age, y	67 ± 10
Male, n (%)	199 (84.7)
Body surface area, m^2^	1.729 ± 0.181
Medical history	
History of MI, n (%)	40 (17.0)
Hypertension, n (%)	182 (77.4)
Hyperlipidemia, n (%)	135 (57.4)
Diabetes mellitus, n (%)	93 (39.6)
Current smoker, n (%)	44 (18.7)
Family history, n (%)	32 (13.6)
Laboratory data	
LDL-chol, mg dL^−1^	87 [72–111]
Creatinine, mg dL^−1^	0.84 [0.72–0.96]
HbA1c, %	6.2 [5.8–6.9]
NT-proBNP, ng L^−1^	124 [59–315]
Hs-cTnI at presentation, ng L^−1^	6 [3–12]
Peak hs-cTnI, ng L^−1^	320 [113–953]
Peak CK, IU L^−1^	101 [69–163]
Peak CK-MB, IU L^−1^	11 [8–15]
Coronary angiography	
Target lesion location; RCA/LAD/LCx	58 (24.7)/146 (62.1)/31 (13.2)
UMI lesion PCI, n (%)	25 (10.6)
OMI lesion PCI, n (%)	25 (10.6)​
SYNTAX score	14 [9–18]
Pre-PCI	
Diameter stenosis, %	73.7 [65.8–81.4]
Lesion length, mm	15.2 [10.9–20.0]
FFR	0.64 [0.50–0.73]
Post-PCI	
Diameter stenosis, %	10.9 [7.9–14.8]
FFR	0.88 [0.83–0.93]
Total stent length, mm	28 [20–40]
Number of stents, n (%)	1 [1–1]
Mean stent diameter, mm	3.25 [3.00–3.50]
CMR indices	
EDV, mL	120.1 [102.7–145.4]
ESV, mL	45.1 [32.8–64.3]
LVMI, g m^−2^	77.3 [69.6–89.5]
EF, %	62.0 [53.8–68.0]
Unrecognized LGE, g	0.0 [0.0–0.0]
Post-PCI LGE, g	0.0 [0.0–9.7]
Increased LGE, g	0.0 [0.0–0.0]

*EDV* end diastolic volume, *EF* ejection fraction, *ESV* end systolic volume, *FFR* fractional flow reserve, *HbA1c* glycated hemoglobin, *Hs-cTnI* high-sensitivity troponin I, *LAD* left anterior descending coronary artery, *LCx* left circumflex coronary artery, *LDL-chol* low density lipoprotein cholesterol, *LGE* late gadolinium enhancement, *LVMI* left ventricular mass index, *NT*-*proBNP* N-terminal pro-B-type natriuretic peptide, *OMI* old myocardial infarction, *PCI* percutaneous coronary intervention, *RCA* right coronary artery.

### UMI detected by CMR

**Figure 2:**
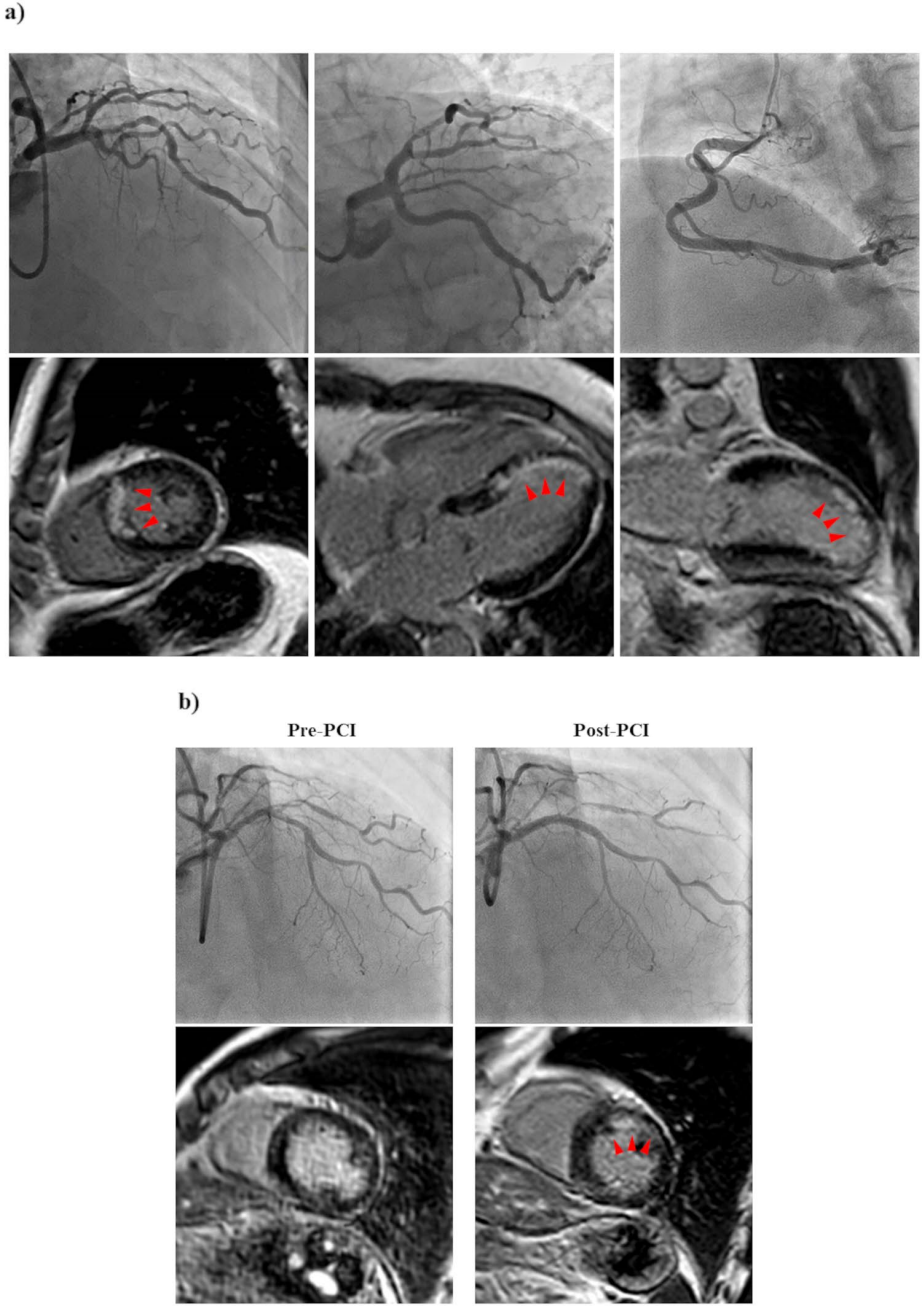
Representative cardiac magnetic resonance images and angiographies (**a**) of a patient with unrecognized myocardial infarction; (**b**) of a patient with periprocedural new occurrence of LGE after PCI.

Examples are shown in Fig. 2a. Median UMI volume was 8.7 (4.0–19.5) g in patients with UMI. The UMIs were predominantly located in the target vessel territories of PCI, accounting for 58.1% of cases. The presence of UMI was significantly associated with FFR and angiographic stenosis severity in the target vessels (UMI (+) vs UMI (−); FFR: 0.59 vs 0.66, P = 0.020; diameter stenosis: 76.7 vs 73.6%, P = 0.033, respectively). Patients with UMI were more likely to be male and have a higher body surface area (BSA), high-sensitivity cardiac troponin (hs-cTnI) levels at baseline, SYNTAX Scores, and prevalence of diabetes mellitus (P = 0.002, P < 0.001, P = 0.045, P < 0.001, P = 0.006, respectively).

### Incidence of PPL and cardiac biomarker elevation

The present study assessed PMI by PPL and cardiac biomarker elevation. In a total of 235 patients in a final analysis, 45 (19.1%) patients presented PPL on post-PCI CMR. Examples are shown in Fig. 2b. The median PPL mass was 3.9 g. Patients with PPL had greater biomarkers release than those without (hs-cTnI: 932 (207–7806) ng L^−1^ vs 285 (110–770) ng L^−1^, P < 0.001; CK-MB: 17 (10–34) U L^−1^ vs 11 (8–13), P < 0.001) in blood samples obtained at an average of 21.0 ± 1.5 h after PCI completion. Post-PCI hs-cTnI elevation ≥ 5 × URL occurred in 169 patients (71.9%), post-PCI hs-cTnI elevation ≥ 70 × URL in 37 patients (15.7%), and post-PCI CK-MB elevation ≥ 10 × URL in 1 patient (0.4%). There was a significant correlation between cardiac biomarker elevation and PPL (hs-cTnI: r = 0.67, P < 0.001, CK-MB: r = 0.55, P < 0.001, respectively), whereas 21.9% of patients with hs-cTnI elevation ≥ 5 × URL and 48.6% with hs-cTnI elevation ≥ 70 × URL showed PPL (Cohen’s kappa = 0.322). Compared to patients without PPL, those with PPL had higher NT-proBNP levels at presentation and were more likely to have UMI in the target vessel territories of PCI (24.4% vs 7.4%, P = 0.002) and multiple stent implantation (P = 0.036).

### Determinants of the presence of UMI and the occurrence of PPL

In univariable and multivariable logistic regression analyses, BSA, the history of DM, SYNTAX Score, LVMI, and lesion stenosis severity were independent predictors of the presence of UMI (OR 11.5, 95% CI 2.53–82.6, P = 0.005; OR 2.28, 95% CI 1.09–4.79, P = 0.030; OR 1.12, 95% CI 1.06–1.20, P < 0.001; OR 1.02, 95% CI 1.00–1.04, P = 0.026, OR 1.03, 95% CI 1.00–1.07, P = 0.039, respectively) (Table 2). Meanwhile, UMI in the target vessel territories and log (NT-proBNP) before the index PCI were independent predictors of the occurrence of PPL (OR 3.77, 95% CI 1.52–9.36, P = 0.004; OR 1.32, 95% CI 1.05–1.65, P = 0.016, respectively), whereas the presence of recognized myocardial infarction in the target area was not significant (OR 1.06, 95% CI 0.38–3.00, P = 0.91) (Table 3).

**Table 2:** Univariate and multivariate logistic regression analysis for parameters that correlated with UMI.

	Univariable analysis			Multivariable analysis Model 1			Multivariable analysis Model 2		
	OR	95% CI	P value	OR	95% CI	P value	OR	95% CI	P value
Age	0.97	0.94–1.00	0.057						
Male	0	0.00–0.00	0.998						
Body surface area	33.0	4.72–230.2	< 0.001	11.5	2.53–82.6	0.005	12.2	1.48–99.8	0.020
DM^†^	2.53	1.29–4.97	< 0.001	2.28	1.09–4.79	0.030	2.28	1.10–4.72	0.027
Current smoker^†^	1.94	0.60–4.17	0.091						
SYNTAX score	1.14	1.07–1.21	< 0.001	1.12	1.06–1.20	< 0.001		Not selected	
Pre-PCI DS	1.05	1.02–1.08	0.003		Not selected		1.03	1.00–1.07	0.039
Pre-PCI FFR	0.21	0.03–1.35	0.10						
Hs-cTnI at presentation	1.00	0.98–1.01	0.49						
LVMI	1.03	1.01–1.05	< 0.001	1.02	1.00–1.04	0.026	1.02	1.01–1.04	0.007
EF	0.97	0.95–0.99	0.012	1.01	0.97–1.04	0.73	1.00	0.96–1.03	0.82

*DM* diabetes mellitus, *DS* diameter stenosis, *EF* ejection fraction, *FFR* fractional flow reserve, *Hs-cTnI* high-sensitivity troponin I, *LVMI* left ventricular mass index, *PCI* percutaneous coronary intervention.

^†^Categorical variables.

**Table 3:** Univariate and multivariate logistic regression analysis for parameters that correlated with PPL.

	Univariable analysis			Multivariable analysis		
	OR	95% CI	P value	OR	95% CI	P value
HT^†^	1.02	0.47–2.24	0.95			
HL^†^	0.65	0.34–1.25	0.20			
DM^†^	1.28	0.67–2.47	0.46			
Current smoker^†^	0.62	0.24–1.56	0.31			
UMI lesion PCI^†^	4.41	1.82–10.7	0.001	3.77	1.52–9.36	0.004
OMI lesion PCI^†^	1.06	0.38–3.00	0.91			
Pre-PCI FFR	0.87	0.12–6.07	0.89			
Mean stent diameter	1.77	0.84–3.72	0.13			
Total stent length	1.01	1.00–1.03	0.15			
Number of implanted stents^†^	2.14	1.04–4.39	0.038	1.68	0.77–3.67	0.19
Total-chol	0.99	0.98–1.00	0.15			
LDL-chol	0.99	0.98–1.00	0.14			
Log (NT-proBNP)	1.38	1.11–1.71	0.004	1.32	1.05–1.65	0.016

*DM* diabetes mellitus, *FFR* fractional flow reserve, *HL* hyperlipidemia, *HT* hypertension, *NT* *proBNP* N-terminal pro-B-type natriuretic peptide, *OMI* old myocardial infarction, *PCI* percutaneous coronary intervention, *UMI* unrecognized MI.

^†^Categorical variables.

### Prognostic value of UMI and PPL

During the follow-up period of 2.2 (1.4–3.0) years, 31 of 235 patients (13.2%) reached the composite endpoint, including 1 cardiovascular death (0.4%), 6 nonfatal MI (2.6%), 3 hospitalization for congestive heart failure (1.3%), 2 stroke (0.9%), and 19 (8.1%) unplanned late revascularization.

**Figure 3:**
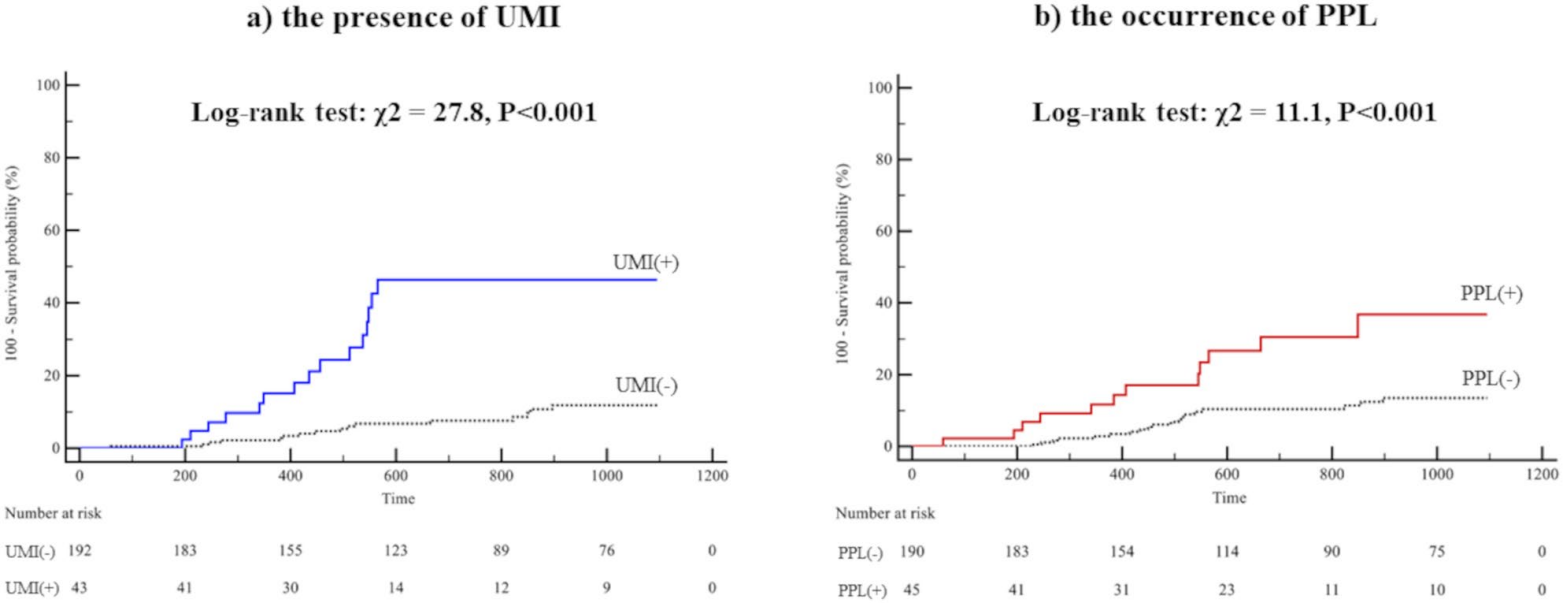
Kaplan–Meier analysis for the incidence of MACE stratified by (**a**) the presence of UMI; (**b**) the occurrence of PPL. Event-free survival was significantly worse in patients with UMI and PPL.

**Figure 4:**
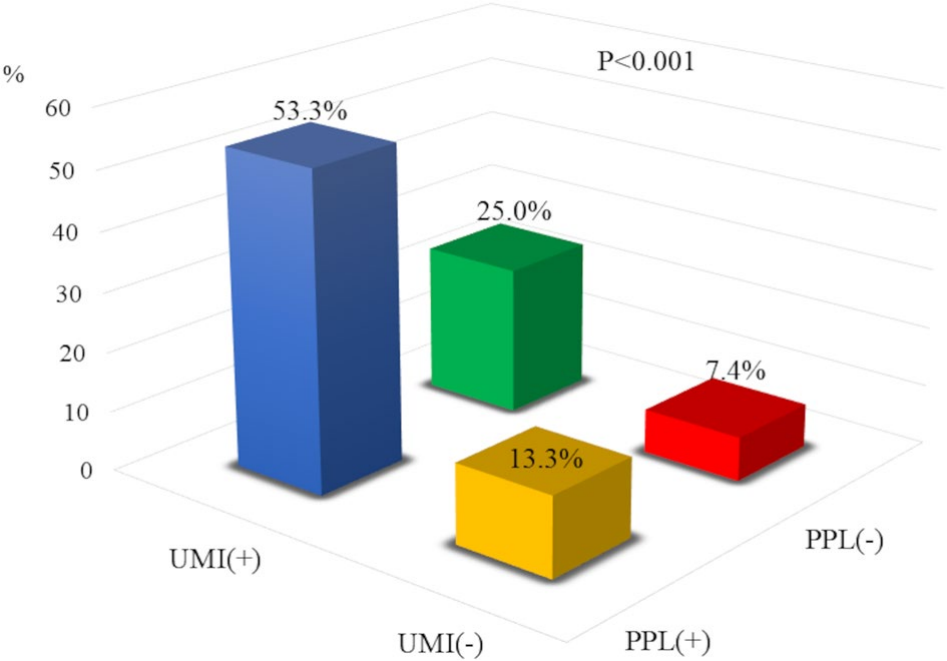
Frequency of MACE stratified into four groups by the presence or occurrence of UMI and PPL. Blue: UMI (+), PPL (+); green: UMI (+), PPL (−); yellow: UMI (−), PPL (+); red: UMI (−), PPL (−). The patients with UMI and PPL showed significantly higher frequency of MACE.

Cox proportional hazard analysis revealed that the presence of UMI and the occurrence of PPL were independent predictors of MACE (Model 1 (adjusted by age and male sex): HR 4.62, 95% CI 2.24–9.54, P < 0.001; HR 2.33, 95% CI 1.11–4.91, P = 0.026; Model 2 (adjusted by EF and Syntax score): HR 4.62, 95% CI 2.23–9.57, P < 0.001; HR 2.33, 95% CI 1.11–4.93, P = 0.026) (Table 4). In contrast, post-PCI hs-cTnI elevation ≥ 5 × URL, hs-cTnI elevation ≥ 70 × URL, and CK-MB elevation ≥ 10 × URL were not significantly associated with MACE (P = 0.10, P = 0.070, P = 0.77, respectively). Kaplan–Meier analysis demonstrated a significantly increased risk of MACE in patients with UMI compared to those without, and the patients with PPL compared to those without (P < 0.001, P < 0.001, respectively) (Fig. 3). When stratified into four groups according to UMI presence and PPL occurrence, the patients with UMI and PPL had a significantly higher incidence of MACE (P < 0.001, Fig. 4).

**Table 4:** Cox proportional-hazard regression analysis of MACE.

	Univariable analysis			Multivariable analysis Model 1			Multivariable analysis Model 2		
	HR	95% CI	P value	HR	95% CI	P value	HR	95% CI	P value
Age	1.02	0.98–1.05	0.39	1.02	0.99–1.06	0.26			
Male sex	2.85	0.68–11.9	0.15	1.78	0.41–7.80	0.44			
HT									
HL									
DM	2.03	1.00–4.15	0.051						
Current smoker, n (%)	0.62	0.22–1.76	0.37						
EF, %	0.96	0.94–0.99	0.004				0.98	0.96–1.01	0.22
Syntax score	1.12	1.06–1.19	< 0.001				1.04	0.98–1.11	0.22
Peak post-PCI hs-cTnI	1.00	1.00–1.00	0.39						
Post-PCI hs-cTnI > 5 × 99%URL	2.21	0.85–5.76	0.10						
Post-PCI hs-cTnI > 70 × 99%URL	2.11	0.94–4.72	0.070						
Peak CK-MB, IU L^−1^	1.00	0.99–1.01	0.86						
Post-PCI LGE volume	1.03	1.00–1.06	0.067						
UMI presence	5.47	2.69–11.1	< 0.001	4.62	2.24–9.54	< 0.001	4.62	2.23–9.57	< 0.001
PPL occurrence	3.21	1.55–6.62	0.002	2.33	1.11–4.91	0.026	2.33	1.11–4.93	0.026

*DM* diabetes mellitus, *EF* ejection fraction, *HL* hyperlipidemia, *Hs-cTnI* high-sensitivity troponin I, *HT* hypertension, *LGE* late gadolinium enhancement, *PCI* percutaneous coronary intervention, *PPL* periprocedural new occurrence or increased volume of LGE in the target territory after revascularization, *UMI* unrecognized myocardial infarction.

## Discussion

The current study evaluated the prevalence or occurrence of UMI and PPL simultaneously using serial DE-CMR examinations and demonstrated the prognostic significance of these entities in CCS patients undergoing elective PCI in the same cohort for the first time.

The major findings of our studies are as follows: (1) the prevalence of UMI was 18.3%, and the occurrence of PPL was 19.1%; (2) UMI was significantly associated with atherosclerotic risk factors, including higher BSA, the prevalence of DM, SYNTAX Score, LVMI, and lesion stenosis; (3) the presence of UMI in the lesion downstream the target vessel was a significant factor to predict PPL; (4) the presence of UMI and the occurrence of PPL were independently associated with worse outcomes after elective PCI, whereas cardiac marker elevation following PCI was not a significant predictor of MACE.

Considering the prognostic impact of UMI and PPL shown in the present study, these two factors might be relevant to outcomes after elective PCI and could play important roles as non-negligible confounding factors of adverse events because these factors are not routinely worked up in the clinical practice.

### Association between UMI and PPL

We present that UMI was significantly associated with atherosclerotic risk factors and that PCI performed for the lesion related to previous UMI was a significant factor in the occurrence of PPL, whereas PCI for lesions related to previously recognized MI was not. The exact mechanism of this finding remains to be determined, since the mechanism of UMI itself has not been fully established. We assume that target lesion plaque instability, stenosis severity, or microvascular dysfunction associated with UMI might at least partly explain the link between UMI and subsequent PPL. Increased atherosclerotic burden is another possible associated factor, as the distal coronary embolization of intracoronary thrombus and/or lipid-core plaque contents contribute to no-reflow phenomenon^13^. Undertreated atherosclerotic factors in the patients with UMI might also contribute to periprocedural myocardial injury presented by PPL. Previous studies reported that modifiable cardiovascular risk factors are strongly associated with the presence of UMI, whereas suspected CAD patients with UMI had a lower rate of optimal or guideline directed medical therapy compared to the patients with clinically recognized MI^1^. Since all the patients enrolled in this study were scheduled for elective PCI, they received guideline-directed medical therapy at the time of the enrollment. However, no secondary preventive therapy was initiated before the study enrollment in patients with UMI, as these patients are clinically unrecognized. Further large prospective studies are needed to test these hypotheses.

### Relationship between UMI/PPL and prognosis after PCI

Importantly, UMI and PPL were independently associated with MACE, suggesting that even a small unrecognized and periprocedural infarction could be associated with MACE when detected as an infarct scar by DE-CMR. When the analysis is exploratorily limited for the MACE subgroup by excluding unplanned late revascularization, UMI and PPL were still found to be independently and significantly associated with worse outcomes (UMI: HR 4.89, 95% CI 1.58–15.2, P = 0.006; PPL: HR 9.83, 95% CI 2.95–32.8, P < 0.001, respectively), although the number of were small.

In accordance with a previous study^9^, we report that PPL might provide better prognostic efficacy than cardiac biomarker elevation. We further show a moderate correlates with post-PCI cardiac biomarker elevations in the present study. Although cardiac biomarkers are easily accessible and available for detecting myocardium damage, it has been reported that post-PCI cardiac biomarker elevation may not reflect the loss of viable myocardium accurately, since post-PCI cardiac biomarker elevation is very sensitive but not specific, particularly for high-sensitivity cardiac troponin^14,15^. In this context, DE-CMR is the gold-standard imaging technique for the detection and quantification of irreversible myocardial injury. Further large prospective studies are required to test our hypothesis for elucidating the mechanisms of the prognostic impact and association of UMI and PPL with worse outcomes.

### Potential clinical implications

It was recently reported that an initial invasive strategy failed to reduce ischemic events and improve survival in patients with CCS compared with an initial conservative strategy^16^, raising the debate on coronary revascularization in CCS. While such studies might favor conservative strategies in CCS, the possible reasons behind these findings should be elucidated before the strict preference of a conservative approach. A variety of patient and lesion characteristics and periprocedural factors have been demonstrated to be associated with worse outcomes in CCS patients undergoing PCI and might confound the results against invasive strategies^17,18^. The result of current study adds to these factors, suggesting that UMI and PPL detected by DE-CMR in patients who underwent elective PCI might provide significant prognostic information over conventional risk stratification. The current guidelines for secondary prevention after MI have focused on nonfatal MI, death, and heart failure hospitalization as key events to initiate the recommended treatment. Patients with UMI are precluded from the recommended aggressive preventive management strategy, leaving them undertreated. Although serial DE-CMR tests are not routinely worked up in clinical practice, and it remains elusive whether any specific intervention or aggressive treatment for patients with UMI and/or PPL improves prognosis after PCI, this substudy suggests UMI and PPL can be used as a risk stratification tool for patients undergoing revascularization since even a minute unrecognized and/or periprocedural infarction were associated with MACE. Further prospective studies are needed to test the hypothesis suggested by this study and identify patients in whom serial CMR examinations provide benefit in routine clinical practice since this hypothesis, based on our study, is merely speculative. It is also warranted to test if the primary intense intervention of conventional risk factors associated with the presence of UMI shown in this study could reduce the development of UMI, since undetected silent ischemia has been prevailingly considered to be the vital risk for adverse events in CCS patients^1,19^.

### Study limitations

The results of the present study should be interpreted with consideration for several significant limitations. This study included a relatively small number of patients from a single center which precluded extensive subgroup or powered multivariable analyses. Due to the small number of events and unadjusted confounders, our results that UMI and PPL were significant prognostic factors independent of conventional risk factors should not be taken as a decisive finding and should be tested in the future studies. Second, rigorous exclusion criteria and the CMR protocol limited the number of study patients and may have resulted in a certain level of selection bias, which may limit the generalizability of the present study. Most of the patients were male and patients with impaired EF were excluded. It should also be noted that, unlike previous studies, this study did not exclude patients with a history of MI to investigate the effects of recognized and unrecognized MI on the occurrence of PPL and subsequent events. Therefore, the possibility of underestimating the presence of UMI cannot be ruled out. Third, partial volume effects and cardiac motion might have affected the results, although the measurement reliability was acceptable for assessing LGE with the current spatial resolution of CMR. Furthermore, although CMR imaging was performed after a median of 12 (7–18) days of PCI, different time windows of CMR should be evaluated in future studies since changes in LGE volume over time might affect the diagnostic accuracy in detecting small periprocedural infarcts. Fourth, the assessment of involved myocardial segments by a coronary stenosis was determined by the coronary anatomy, and no objective method was applied, although there are no universally accepted criteria for this purpose. Fifth, T2 STIR was not performed in the present study, which may be useful to differentiate periprocedural infarcts after PCI. Finally, it must be taken into consideration that the potentially poor symptom recognition by patients with UMI may also contribute to lower events rates during the follow-up period, although this hypothetical possibility is more likely to result in an increase in the MACE risk of patients with UMI.

## Conclusions

UMI and PPL evaluated by serial DE-CMR examinations before and after elective PCI were significantly associated with worse outcomes independent of conventional risk factors in patients with CCS. UMI and PPL detected by DE-CMR might provide additional potential insight for the risk stratification of patients undergoing elective PCI in this population.

### Supplementary Information

Supplementary Information.

## Data Availability

The datasets used and/or analyzed during the current study are available from the corresponding author on reasonable request.

## Abbreviations

CCS: Chronic coronary syndrome
CK-MB: Creatine kinase-MB
DE-CMR: Delayed enhancement cardiac magnetic resonance imaging
EF: Ejection fraction
Hs-cTnI: High-sensitivity cardiac troponin I
LGE: Late gadolinium enhancement
MACE: Major adverse cardiovascular events
PCI: Percutaneous coronary intervention
PMI: Periprocedural myocardial injury
PPL: Periprocedural new occurrence or increased volume of late gadolinium enhancement
UMI: Unrecognized myocardial infarction
