# Supplementary material for: Prognostic implications of unrecognized myocardial infarction and periprocedural myocardial injury on cardiac magnetic resonance imaging in patients with chronic coronary syndrome
Source: Sci Rep. 2023 Aug 21;13:13567. doi: 10.1038/s41598-023-40883-2 (PMC10442331; doi:10.1038/s41598-023-40883-2)
Supplement: Supplementary file 1 — Supplementary Information. [file 41598_2023_40883_MOESM1_ESM.docx]

**Prognostic Implications of Unrecognized Myocardial Infarction and Periprocedural Myocardial Injury on Cardiac Magnetic Resonance Imaging in Patients with Chronic Coronary Syndrome**

Yoshihisa Kanaji, MD, PhD^1,2^, Masahiro Hoshino, MD^1^, Masahiro Hada, MD^1^, Ilke Ozcan, MD^2^, Tomoyo Sugiyama, MD, PhD^1^, Kazuki Matsuda, MD^1^, Kodai Sayama, MD^1^, Kai Nogami, MD^1^, Tatsuhiro Nagamine, MD^1^, Yun Teng, MD^1^, Toru Misawa, MD^1^, Makoto Araki, MD^3^, Eisuke Usui, MD^3^, Tadashi Murai, MD, PhD^1^, Taishi Yonetsu, MD^3^, Tetsuo Sasano, MD. PhD^3^, and Tsunekazu Kakuta, MD, PhD^1^

^1^Division of Cardiovascular Medicine, Tsuchiura Kyodo General Hospital, Ibaraki, Japan

^2^Department of Cardiovascular Medicine, Mayo Clinic, Rochester, MN, USA

^3^Department of Cardiovascular Medicine, Tokyo Medical and Dental University, Tokyo, Japan

All authors take responsibility for all aspects of the reliability and freedom from bias of the data presented and their discussed interpretation.

**Address for correspondence:**

Tsunekazu Kakuta, MD, PhD

Department of Cardiovascular Medicine,

Tsuchiura Kyodo General Hospital

4-1-1, Otsuno, Tsuchiura, Ibaraki 300-0028, Japan

E-mail:[kaz@joy.email.ne.jp](mailto:kaz@joy.email.ne.jp) Tel:+81-29-830-3711 Fax:+81-29-846-3721

**Supplemental material 1. CMR image acquisition**

Cardiac gating and heart rate recording were achieved by the vector-cardiogram device. Cine-CMR was performed using a retrospectively gated steady-state free precession sequence. Twelve short axis slices of the left ventricle (LV) were acquired from the apex to the base. The cine-CMR parameters were as follows: repetition time/echo time 4.1 ms/1.4 ms, slice thickness 6 mm, flip angle 55°, field of view 350×350 mm2, matrix size 128×128, and number of phases per cardiac cycle 20. LV mass and volumes were calculated according to the Simpson’s rule using CMR data. Thereafter, gadolinium contrast was injected intravenously at a total dose of 0.10 mmol/kg. Fifteen minutes following injection, LGE images were acquired in the same planes as cine images and imaging parameters were repetition time/echo time 3.8 ms/1.28 ms, flip angle 15°, field of view 350×350 mm2, acquisition matrix 200×175, number of phases per cardiac cycle 20, slice thickness 8 mm.

**Supplemental table 1. The baseline clinical characteristics**

|  | Total, N=235 | UMI (-), N=192 | UMI (+), N=43 | P value | PPL (-), N=190 | PPL (+), N=45 | P value |
| --- | --- | --- | --- | --- | --- | --- | --- |
| ***Demographics*** |  |  |  |  |  |  |  |
| Age, y | 67 ±10 | 67 ±10 | 64 ±12 | 0.055 | 66 ±10 | 68 ±12 | 0.27 |
| Male, n (%) | 199 (84.7) | 156 (81.3) | 43 (100) | 0.002 | 160（84.2） | 39（86.7） | 0.68 |
| Body surface area, m^2^ | 1.729 ±0.181 | 1.709 ±0.182 | 1.820 ±0.149 | <0.001 | 1.731 ±0.184 | 1.720 ±0.171 | 0.72 |
| ***Medical history*** |  |  |  |  |  |  |  |
| History of MI, n (%) | 40 (17.0) | 38 (19.8) | 2 (4.7) | 0.017 | 29 (15.3) | 11 (24.4) | 0.14 |
| Hypertension, n (%) | 182 (77.4) | 150 (78.1) | 32 (74.4) | 0.60 | 147 (77.4) | 35 (77.8) | 0.95 |
| Hyperlipidemia, n (%) | 135 (57.4) | 111 (57.8) | 24 (55.8) | 0.81 | 113 (59.5) | 22 (48.9) | 0.24 |
| Diabetes mellitus, n (%) | 93 (39.6) | 68 (35.4) | 25 (58.1) | 0.006 | 73 (38.4) | 20 (44.4) | 0.46 |
| Current smoker, n (%) | 44 (18.7) | 32 (16.7) | 12 (27.9) | 0.088 | 38 (20.0) | 6 (13.3) | 0.30 |
| Family history, n (%) | 32 (13.6) | 27 (14.1) | 5 (11.6) | 0.67 | 24 (12.6) | 8 (17.8) | 0.37 |
| ***Prescription at admission*** |  |  |  |  |  |  |  |
| Statin, n (%) | 211 (89.8) | 173 (90.1) | 38 (88.4) | 0.74 | 171 (90.0) | 40 (88.9) | 0.83 |
| ACE-I or ARB, n (%) | 161 (68.5) | 132 (68.8) | 29 (67.4) | 0.87 | 126 (66.3) | 35 (77.8) | 0.14 |
| β-blocker, n (%) | 156 (66.4) | 129 (667.2) | 27 (62.8) | 0.58 | 125 (65.8) | 31 (68.9) | 0.69 |
| Calcium antagonist, n (%) | 93 (39.6) | 78 (40.6) | 15 (34.9) | 0.49 | 76 (40.0) | 17 (37.8) | 0.78 |
| **Laboratory data** |  |  |  |  |  |  |  |
| T-chol, mg dL^-1^ | 165 [144 - 187] | 164 [144 - 186] | 166 [146 - 195] | 0.77 | 166 [145 - 189] | 151 [141 - 175] | 0.087 |
| LDL-chol, mg dL^-1^ | 87 [72 - 111] | 86 [71 - 110] | 92 [78 - 115] | 0.42 | 90 [74 - 115] | 83 [67 - 101] | 0.099 |
| HDL-chol, mg dL^-1^ | 47 [41 - 56] | 47 [41 - 56] | 47 [40 - 55] | 0.81 | 47 [42 - 56] | 46 [40 - 58] | 0.90 |
| TG, mg dL^-1^ | 129 [92 - 174] | 131 [93 - 175] | 125 [89 - 163] | 0.45 | 129 [95 - 178] | 128 [85 - 159] | 0.34 |
| Creatinine, mg dL^-1^ | 0.84 [0.72 - 0.96] | 0.84 [0.72 - 0.95] | 0.86 [0.75 - 1.04] | 0.12 | 0.84 [0.72 - 0.95] | 0.86 [0.73 - 1.01] | 0.35 |
| eGFR, ml min^-1^ 1.73m^-2^ | 67.6 [58.8 - 78.9] | 67.4 [59.5 - 78.5] | 69.0 [54.9 - 79.6] | 0.94 | 68.1 [59.8 - 79.2] | 65.2 [52.9 - 76.8] | 0.14 |
| HbA1c, % | 6.2 [5.8 - 6.9] | 6.1 [5.8 - 6.8] | 6.6 [5.9 - 7.3] | 0.013 | 6.2 [5.8 - 6.8] | 6.3 [5.8 - 7.1] | 0.58 |
| NT-proBNP, ng L^-1^ | 124 [59 - 315] | 120 [56 - 303] | 187 [77 - 393] | 0.21 | 107 [53 - 240] | 234 [113 - 723] | 0.002 |
| Hs-cTnI at presentation, ng L^-1^ | 6 [3 - 12] | 6 [3 - 12] | 9 [4 - 16] | 0.045 | 6 [3 - 12] | 7 [5 - 13] | 0.22 |
| Peak hs-cTnI, ng L^-1^ | 320 [113 - 953] | 338 [125 - 930] | 240 [82 - 1050] | 0.46 | 285 [110 - 770] | 932 [207 - 7806] | <0.001 |
| Post-PCI hs-cTnI ˃ 5×99%URL, n (%) | 169 (71.9%) | 143 (74.5) | 26 (60.5) | 0.090 | 132 (69.5) | 37 (82.2) | 0.099 |
| Post-PCI hs-cTnI ˃ 70×99%URL, n (%) | 37 (15.7) | 30 (15.6) | 7 (16.3) | 1 | 19 (10.0) | 18 (40.0) | <0.001 |
| peak CK, IU L^-1^ | 101 [69 - 163] | 99 [68 - 162] | 103 [81 - 163] | 0.43 | 99 [68 - 153] | 110 [78 - 288] | 0.085 |
| peak CK-MB, IU L^-1^ | 11 [8 - 15] | 11 [8 - 15] | 12 [8 - 17] | 0.64 | 11 [8 - 13] | 17 [10 - 34] | <0.001 |
| hs-CRP, mg dL^-1^ | 0.060 [0.030 - 0.150] | 0.060 [0.030 - 0.138] | 0.070 [0.040 - 0.150] | 0.17 | 0.050 [0.030 - 0.140] | 0.060 [0.038 - 0.175] | 0.13 |
| ***Coronary angiography*** |  |  |  |  |  |  |  |
| Target lesion location; RCA/LAD/LCx | 58 (24.7)/146 (62.1)/31 (13.2) | 49 (25.5)/120 (62.5)/23 (12.0) | 9 (20.9)/26 (60.5)/8 (18.6) | 0.47 | 44 (23.2)/121 (63.7)/25 (55.6) | 14 (31.1)/25 (55.6)/6 (13.3) | 0.52 |
| UMI lesion PCI, n (%) | 25 (10.6) | 0 (0) | 25(58.1) | <0.001 | 14 (7.4) | 11 (24.4) | 0.002 |
| OMI lesion PCI, n (%) | 25 (10.6)​ | 25 (13.0)​ | 0 (0)​ | <0.011​ | 20 (10.5)​ | 5 (11.1)​ | 1​ |
| SYNTAX score | 14 [9 - 18] | 13 [9 - 17] | 18 [13 - 23] | <0.001 | 14 [9 - 18] | 15 [9 - 21] | 0.53 |
| **Pre-PCI** |  |  |  |  |  |  |  |
| Minimum lumen diameter, mm | 0.78 [0.55 - 0.99] | 0.78 [0.58 - 1.00] | 0.76 [0.28 - 0.90] | 0.039 | 0.78 [0.58 - 1.00] | 0.76 [0.28 - 0.90] | 0.32 |
| Reference diameter, mm | 2.82 [2.59 - 3.15] | 2.83 [2.59 - 3.20] | 2.76 [2.59 - 3.00] | 0.41 | 2.83 [2.59 - 3.20] | 2.76 [2.59 - 3.00] | 0.95 |
| Diameter stenosis, % | 73.7 [65.8 - 81.4] | 73.6 [65.4 - 80.7] | 76.7 [67.4 - 89.5] | 0.033 | 73.6 [65.4 - 80.7] | 76.7 [67.4 - 89.5] | 0.51 |
| Lesion length, mm | 15.2 [10.9 - 20.0] | 14.5 [10.9 - 19.2] | 16.1 [10.9 - 23.4] | 0.17 | 14.5 [10.9 - 19.2] | 16.1 [10.9 - 23.4] | 0.63 |
| FFR | 0.64 [0.50 - 0.73] | 0.66 [0.53 - 0.74] | 0.59 [0.40 - 0.69] | 0.020 | 0.66 [0.53 - 0.74] | 0.59 [0.40 - 0.69] | 0.96 |
| **Post-PCI** |  |  |  |  |  |  |  |
| Minimum lumen diameter, mm | 2.94 [2.59 - 3.27] | 2.92 [2.55 - 3.25] | 3 [2.80 - 3.31] | 0.17 | 2.96 [2.62 - 3.28] | 2.88 [2.53 - 3.13] | 0.38 |
| Reference diameter, mm | 3.32 [3.00 - 3.63] | 3.31 [2.95 - 3.65] | 3.37 [3.20 - 3.57] | 0.28 | 3.32 [3.02 - 3.65] | 3.23 [2.94 - 3.57] | 0.57 |
| Diameter stenosis, % | 10.9 [7.9 - 14.8] | 11.0 [8.0 - 14.8] | 10.1 [7.9 - 14.7] | 0.42 | 10.7 [7.5 - 14.6] | 12.0 [9.4 - 16.0] | 0.14 |
| FFR | 0.88 [0.83 - 0.93] | 0.88 [0.83 - 0.93] | 0.85 [0.81 - 0.92] | 0.16 | 0.88 [0.83 - 0.92] | 0.86 [0.83 - 0.94] | 0.83 |
| Total stent length, mm | 28 [20 - 40] | 28 [20 - 38] | 38 [25 - 62] | <0.001 | 28 [20 - 39] | 30 [23 - 47] | 0.25 |
| Number of stents, n (%) | 1 [1 - 1] | 1 [1 - 1] | 1 [1 - 2] | <0.001 | 1 [1 - 1] | 1 [1 - 2] | 0.036 |
| Mean stent diameter, mm | 3.25 [3.00 - 3.50] | 3.25 [3.00 - 3.50] | 3.00 [3.00 - 3.50] | 0.42 | 3.25 [3.00 - 3.50] | 3.50 [3.00 - 3.50] | 0.12 |
| ***CMR indices*** |  |  |  |  |  |  |  |
| EDV, mL | 120.1 [102.7 - 145.4] | 118.6 [101.4 - 138.7] | 136.2 [116.7 - 178.3] | 0.002 | 119.8 [102.8 - 144.9] | 122.4 [99.8 - 147.1] | 0.95 |
| ESV, mL | 45.1 [32.8 - 64.3] | 43.8 [32.5 - 59.2] | 57.9 [38.1 - 86.4] | 0.002 | 45.2 [33.0 - 64.4] | 45.1 [32.4 - 64.2] | 0.95 |
| LVMI, g m^-2^ | 77.3 [69.6 - 89.5] | 75.5 [68.2 - 87.6] | 85.8 [76.4 - 110.0] | 0.001 | 77.3 [68.5 - 89.2] | 78.2 [70.9 - 95.1] | 0.18 |
| EF, % | 62.0 [53.8 - 68.0] | 62.2 [55.2 - 68.2] | 58.1 [45.5 - 64.8] | 0.032 | 61.6 [53.9 - 68.4] | 62.8 [53.5 - 66.3] | 0.83 |
| Pre-PCI LGE, g | 0.0 [0.0 – 2.9] | 0.0 [0.0 - 0.0] | 8.7 [4.0 - 19.5] | <0.001 | 0.0 [0.0 - 6.2] | 3.4 [0.0 - 8.8] | 0.029 |
| Unrecognized LGE, g | 0.0 [0.0 - 0.0] | 0.0 [0.0 - 0.0] | 8.7 [4.0 - 19.5] | <0.001 | 0.0 [0.0 - 0.0] | 0.0 [0.0 - 4.3] | 0.004 |
| UMI presence, n (%) | 43 (18.3) | - | - | - | 28 (14.7) | 15 (33.3) | 0.004 |
| Post-PCI LGE, g | 0.0 [0.0 – 9.7] | 0.0 [0.0 – 4.3] | 13.0 [4.5 - 19.9] | <0.001 | 0.0 [0.0 – 4.7] | 9.0 [3.4 – 17.3] | <0.001 |
| Increased LGE, g | 0.0 [0.0 - 0.0] | 0.0 [0.0 - 0.0] | 0.0 [0.0 - 3.1] | 0.003 | 0.0 [0.0 - 0.0] | 3.9 [2.6 - 6.1] | <0.001 |
| PPL occurrence, n (%) | 45 (19.1) | 30 (15.6) | 15 (34.9) | 0.004 | - | - | - |

ACE -I: angiotensin-converting enzyme inhibitor; ARB: angiotensin receptor blocker; cTnI: cardiac troponin I; CMR: cardiac magnetic resonance imaging; EDV: end diastolic volume; EF: ejection fraction; eGFR: estimated glomerular filtration rate; ESV: end systolic volume; FFR: fractional flow reserve; HbA1c: glycated hemoglobin; HDL-chol: high density lipoprotein cholesterol; hs-CRP: high sense c-reactive protein; Hs-cTnI: high-sensitivity troponin I; LAD: left anterior descending coronary artery; LCx: left circumflex coronary artery; LDL-chol: low density lipoprotein cholesterol; LGE: late gadolinium enhancement; LVM: left ventricular mass; LVMI: left ventricular mass index; MI: myocardial infarction; NT-proBNP: N-terminal pro-B-type natriuretic peptide; OMI: old myocardial infarction; PCI: percutaneous coronary intervention; PPL: periprocedural new or increased late gadolinium enhancement; RCA: right coronary artery; TG: triglyceride; UMI: unrecognized myocardial infarction
